# Discriminating Malignant from Benign Testicular Masses Using Multiparametric Magnetic Resonance Imaging—A Prospective Single-Center Study

**DOI:** 10.3390/jcm13154390

**Published:** 2024-07-26

**Authors:** Peter Törzsök, Susanne Deininger, Michael Abenhardt, David Oswald, Lukas Lusuardi, Christian Deininger, Rosemarie Forstner, Matthias Meissnitzer, Herwig Brandtner, Stefan Hecht

**Affiliations:** 1 Department of Urology and Andrology, Salzburg University Hospital, Paracelsus Medical University, 5020 Salzburg, Austria; torzsok.peter@gmail.com (P.T.); m.abenhardt@salk.at (M.A.); d.oswald@salk.at (D.O.); l.lusuardi@salk.at (L.L.); 2 Faculty of Health and Sport Sciences, Széchenyi István University, 9026 Győr, Hungary; 3 Department of Orthopedics and Traumatology, Salzburg University Hospital, Paracelsus Medical University, 5020 Salzburg, Austria; c.deininger@salk.at; 4 Institute of Tendon and Bone Regeneration, Paracelsus Medical University, 5020 Salzburg, Austria; 5 Department of Radiology, Paracelsus Medical University, 5020 Salzburg, Austria; r.forstner@salk.at (R.F.); m.meissnitzer@salk.at (M.M.); h.brandtner@salk.at (H.B.); s.hecht@salk.at (S.H.)

**Keywords:** testicular cancer, multiparametric MRI, diffusion-weighted imaging, dynamic contrast-enhanced MRI

## Abstract

**Objective**: The objective of this study was to prospectively assess the extent to which magnetic resonance imaging (MRI) can differentiate malignant from benign lesions of the testis. **Materials and Methods**: All included patients underwent multiparametric testicular MRI, including diffusion-weighted imaging (DWI) and subtraction dynamic contrast-enhanced (DCE) magnetic resonance imaging (MRI). Subsequently, all patients underwent a histopathological examination via orchiectomy or testicular biopsy/partial resection. The Kolmogorov–Smirnov test, *t*-test, Mann–Whitney U test, Fisher’s exact test, and logistic regression were applied for statistical analysis. **Results**: We included 48 male patients (median age 37.5 years [range 18–69]) with testicular tumors. The median tumor size on MRI was 2.0 cm for malignant tumors and 1.1 cm for benign tumors (*p* < 0.05). A statistically significant difference was observed for the type (type 0-III curve, *p* < 0.05) and pattern of enhancement (homogeneous, heterogeneous, or rim-like, *p* < 0.01) between malignant and benign tumors. The minimum apparent diffusion coefficient (ADC) value was 0.9 for benign tumors and 0.7 for malignant tumors (each ×10^3^ mm^2^/s, *p* < 0.05), while the mean ADC was 0.05. The mean ADC value was significantly lower for malignant tumors; the mean ADC value was 1.1 for benign tumors and 0.9 for malignant tumors (each ×10^3^ mm^2^/s, *p* < 0.05). The sensitivity, specificity, positive predictive value, and negative predictive value of multiparametric MRI for differentiating malignant from benign testicular lesions were 94.3%, 76.9%, 91.7%, and 83.3%, respectively. The surgical procedures performed included orchiectomy (*n* = 33; 71.7%) and partial testicular resection (*n* = 11; 23.9%). Histopathology (HP) revealed malignancy in 35 patients (72.9%), including 26 with seminomas and 9 with non-seminomatous germ cell tumors (NSGCTs). The HP was benign in 13 (27.1%) patients, including 5 with Leydig cell tumors. **Conclusions**: Malignant and benign tumors differ in MRI characteristics in terms of the type and pattern of enhancement and the extent of diffusion restriction, indicating that MRI can be an important imaging modality for the accurate diagnosis of testicular lesions.

## 1. Introduction

Testicular cancer is the most common solid neoplasia in men between 15 and 35 years of age [1]. The vast majority of testicular cancers are germ cell neoplasms (GCNs, 90–95%). For the past few decades, the incidence has been rising, particularly in high-income countries [2,3,4,5]. GCNs can be further divided into seminomas and non-seminomatous germ cell tumors (NSGCTs), which include immature or mature teratomas, embryonal carcinomas, yolk sac tumors, choriocarcinomas, or mixed carcinomas with multiple histological subtypes [6]. The age peak for the occurrence of non-seminomas is between 35 and 39 years, while for seminomas it is between 25 and 29 years [7]. Leydig cell tumors (LCTs) are the most prevalent benign tumors [8]. Confirmed risk factors for malignant testicular neoplasms include cryptorchidism, impaired fertility, hypospadia, positive family history, a history of testicular tumor, and ethnicity [2,6]. For risk factors such as cannabis consumption and smoking, the data are unclear [9]. There is even debate about whether a high BMI is protective here [7].

Certain testicular lesions can be detected through palpation, while others are often discovered incidentally during medical evaluations. Typically, around 80% of non-palpable testicular lesions are usually benign [10,11], while palpable masses are more likely to be malignant [12].

Ultrasound (US) of the testis is the imaging modality of choice for the assessment of testicular lesions. The guidelines on testicular cancer set forth by the European Association of Urology (EAU) suggest the use of a high-frequency ultrasound probe (>10 Megahertz) for testicular imaging to examine both the lesion’s location (intra- or extra-testicular) and its size [2]. The evaluation of the contralateral testis is equally important for excluding the presence of lesions or predisposing factors associated with germ cell neoplasia in situ (GCNIS). A sensitivity of up to 98% and a specificity of up to 99.8% for the diagnosis of testicular malignancy are generally high for standard US [13]. The inclusion of extra US techniques, like real-time or shear wave elastography (SWE) or contrast media enhanced-US (CEUS), has proven to enhance the diagnostic accuracy of US and aid in distinguishing between malignant and benign results [14,15]. As part of a meta-analysis, Tufano et al., 2021 demonstrated an accuracy of 96% for CEUS in detecting malignant masses, with a sensitivity of 86% and a specificity of 87% [16]. Nonetheless, categorizing lesions as malignant or benign using only US can be challenging, especially for small lesions [17]. Additional non-invasive diagnostic methods are needed to enable testis-sparing methods such as active surveillance or partial orchiectomy, especially for benign lesions.

As per the EAU guidelines, until now, the use of MRI has been advised only when “US is inconclusive”, for planning testis-sparing operations, for differentiating between intra- and extra-testicular lesions, and for “characterizing intratesticular masses” [2,11,18,19,20]. According to a previous study conducted by our group, MRI revealed a sensitivity of 85.7%, a specificity of 72.8%, a positive predictive value of 52.1%, and a negative predictive value of 93.7%, although this study involved a heterogeneous population from six academic centers [21].

This study aimed to prospectively investigate the significance of multiparametric MRI of the scrotum in patients referred for surgical treatment of suspected testicular tumors based on final histopathological specimens.

## 2. Materials and Methods

### 2.1. Study Population

**Figure 1:**
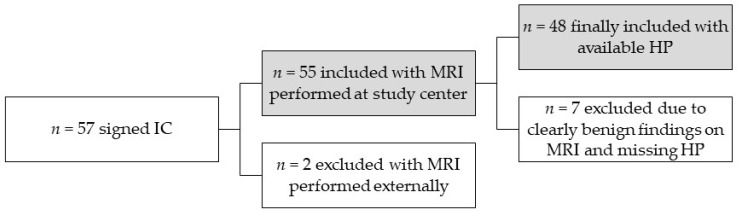
Overview of the included and excluded patients (IC = informed consent, HP = histopathology, MRI = magnetic resonance imaging).

This was a prospective single-institution study approved by the local institutional review board and conducted in accordance with the Declaration of Helsinki. Study inclusion was conditional upon written patient consent and only after histopathological examination, achieved through orchiectomy or testicular biopsy/partial resection following the testicular MRI. Inclusion was limited to patients who underwent testicular MRI at our institution, with external imaging excluded. The eligibility criteria included (1) legal age; (2) ability and willingness to participate; (3) an US diagnosis of an indeterminate (palpable or non-palpable) testicular lesion; (4) a urologic recommendation for surgical intervention; (5) no contraindications to multiparametric MRI, such as implants, kidney diseases, or heart diseases; and (6) a time interval between US, MRI, and surgery of less than 6 weeks. Pathologists who were blinded to the MRI results made the final diagnosis. An overview of the included and excluded patients can be found in Figure 1.

The following patient characteristics were analyzed: age, medical history (smoking history, presence of symptoms, drug abuse status, testicular tumor status, cryptorchism status, and testicular/scrotal operation status), type of diagnosis, physical examination findings (patient’s weight, height, and body mass index [BMI]), US findings (tumor size and site on US), blood test before surgery (tumor markers human chorionic gonadotropin [βHCG], alpha-fetoprotein [AFP], lactate dehydrogenase [LDH], placental alkaline phosphatase [PLAP]), sex hormones (luteinizing hormone [LH], follicle stimulation hormone [FSH], and testosterone), surgical information (duration of the operation, type of surgery, or fresh frozen section [FFS] procedure), definitive histopathology (malignant or benign histology, T-Status, vascular (V) or lymphatic (L) invasion, tumor size, rete testis infiltration, ipsilateral or contralateral testicular intraepithelial neoplasia [TIN], and congruity of FFS with final histology), clinical stage (I–III), and subsequent therapy (conservative, radiotherapy, and/or chemotherapy).

### 2.2. Image Acquisition

All MRI scans were performed on a 3 T Philips Achieva MRI scanner (Philips Medical Systems International B.V., Veenpluis 6, 5684 PC Best, The Netherlands) using a circular surface coil. Patients were placed in the supine position with the penis placed cranially. The testicles were padded with cloths, and the legs were stabilized on the sides with sandbags to avoid motion artifacts and to ensure a comparable distance of both testicles from the coil. Based on current data for the characterization of testicular tumors and based on the current recommendations of the European Society of Urogenital Radiology (ESUR) Scrotal and Penile Imaging Working Group [22], a standardized examination protocol was used, consisting of the following sequences: axial T1WI (3 mm slice thickness, no intersection gap), axial T2WI+  coronal T2WI (3 mm slice thickness, no intersection gap), coronal PD SPAIR (2.5 mm slice thickness, no intersection gap), axial DWI (3 mm slice thickness, no intersection gap), and b values of 0, 450, and 900 s/mm^2^. The highest b value of 900 s/mm^2^ was used for the calculation of the apparent diffusion coefficient (ADC). For subtraction DCE-MRI, a coronal 3D fast-field-echo sequence (2 mm slice thickness, 1 mm overlapping sections) was used. Eight consecutive imaging sets, each with a duration of 60 s, were acquired 15 s after a bolus injection (1–2 mL/s) of contrast agent. Imaging acquisition was continued for 8 min to evaluate washout.

### 2.3. Image Analysis

**Figure 2:**
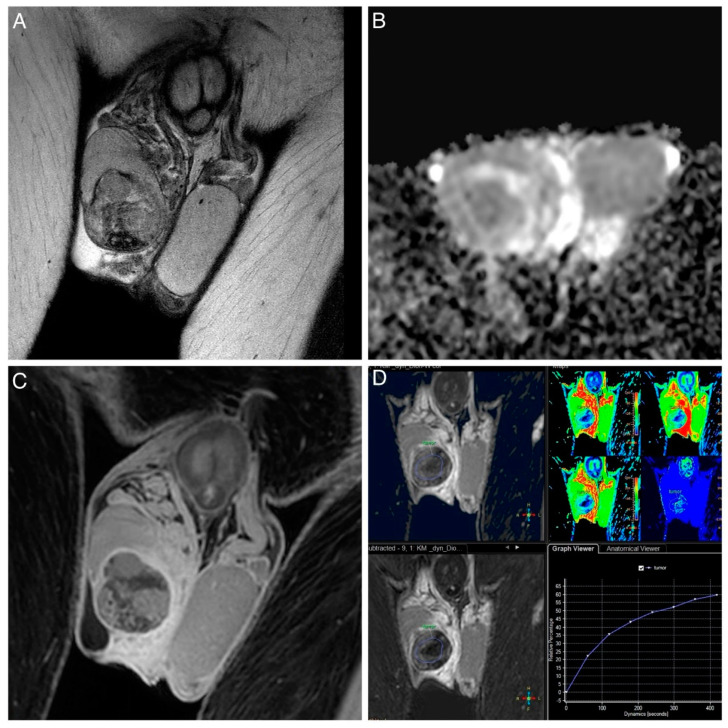
NSGCT in a 23-year-old man. (**A**) T2W image of a heterogeneous right-sided testicular tumor with a small hydrocele. (**B**) ADC map with inhomogeneous diffusion values. (**C**) T1W image after contrast application showing heterogeneous enhancement. (**D**) Perfusion image with a type I curve (progressive pattern; cool color indicates regions with relative decreased blood flow (BF), and warm color indicates regions with relative increased BF).

**Figure 3:**
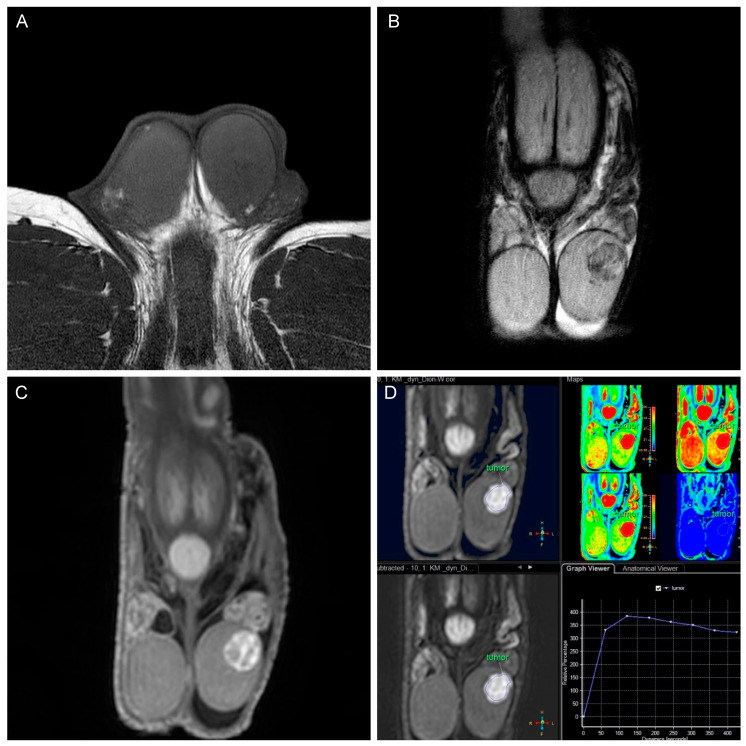
Leydig cell tumor in a 39-year-old man. (**A**,**B**) T1W isointense and T2W hypointense small tumor of the left testicle. Hydrocele. (**C**) T1W image after contrast application showing vivid enhancement. (**D**) Perfusion image with a type 3 curve (strong early enhancement, washout pattern; cool color indicates regions with relative decreased blood flow (BF), and warm color indicates regions with relative increased BF).

All MRIs were evaluated by two radiologists (HS and HB) who were blinded to all clinical data and had 9 and 4 years of experience in urogenital MRI. The following conventional MRI features of the testicular tumors were evaluated on a stationary PACS system (deep unity diagnostics): tumor size and location (intra- or extra-testicular); T2-hypointensity (signal intensity compared to the normal testicle parenchyma); cystic changes (areas of hyperintensity on T2-weighted images representing cyst or necrosis); intratumoral septa (band-like structures of low signal intensity on T2-weighted images enhanced after gadolinium administration); T1 hyperintensity (areas of hyperintensity on T1-weighted images representing hemorrhage); and patterns of enhancement (homogeneous, inhomogeneous, or rim-like). DWI and subtraction DCE-MRI data were transferred to the Philips post-processing workstation (IntelliSpace version 12.1.7) to analyze the mean ADC values, minimum ADC values, and type of contrast enhancement (none or type I–III curve, according to Tsili et al. [23]). The ADC maps were automatically generated from DWI data. Taking the hyperintense signal area of the T2-weighted image and DWI as a reference, regions of interest (ROIs) were drawn on images with b-values of 900 s/mm^2^ or 50 s/mm^2^ (depending on which images displayed the lesion more clearly). The size of the ROIs was variable, as they were drawn as large as possible and depended on the lesion size in a homogeneous low ADC region (avoiding hemorrhage and cystic areas). Similarly, the ROIs were manually placed in the largest enhancing portion of the tumor to generate time–intensity curves (as shown in Figure 2 and Figure 3).

### 2.4. Statistics

All continuous variable data were checked for a normal distribution (normality test: Kolmogorov–Smirnov with Lilliefors significance correction, type I error = 10%). Continuous variables with normally distributed data were compared using a *t*-test for independent samples. For comparisons of continuous variables without normally distributed data and variables measured on ordinal scales, the exact Mann–Whitney U test was used. Dichotomous variables were compared using Fisher’s exact test, and the other categorical variables were compared using the exact chi-square test (including provision of adjusted residuals).

Two-sided 95% confidence intervals (CIs) were determined using parametric, non-parametric, and Clopper-Pearson approaches.

The influence of age, BMI, size of the testicle on MR, and TU size on MR on the occurrence of misdiagnoses was investigated using logistic regression analyses.

Since the type I error was not adjusted for multiple testing, the results of inferential statistics are descriptive only and the use of the term “significant” in the description of the study results always reflects only a local *p* < 0.05, but not an error probability below 5%. Statistical analyses were performed using the open-source R statistical software package, version 4.2.3 (The R Foundation for Statistical Computing, Vienna, Austria).

## 3. Results

### 3.1. Epidemiology and Distribution of Histopathology

In total, 48 patients with a median age of 37.5 years (range 18–69) were included. In total, 31.9% were non-smokers, and 6.8% regularly consumed cannabis. A total of 4.2% had a history of testicular tumors, and 14.6% had a history of undescended testis. In total, 34.8% of the patients had positive tumor markers, and 7.3% had a preoperative testosterone deficiency. The patients’ basic demographics can also be found in Table S1.

**Figure 4:**
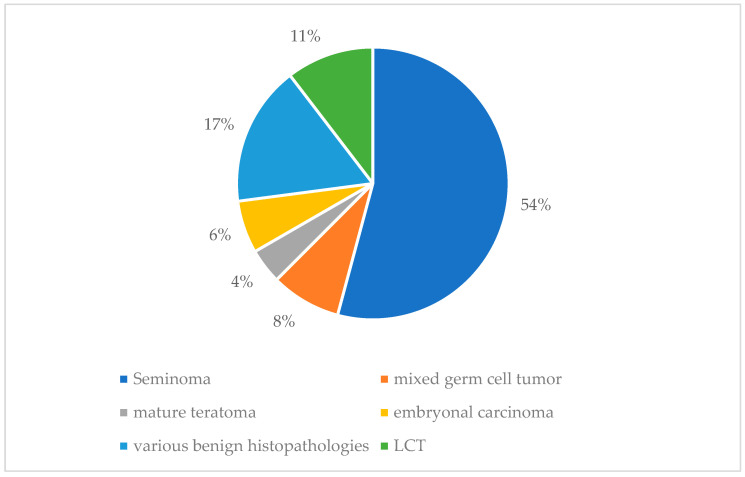
Distribution of histopathology (*n* = 48) according to the World Health Organization (WHO) 5th edition 2022 classification of testicular tumors [24]. NSGCT = non-seminomatous germ cell tumor (*n* = 9), including mixed germ cell tumors (*n* = 4), pure embryonal carcinomas (*n* = 3), and mature teratomas (*n* = 2). Seminoma (*n* = 26; specific histological subtypes not provided); benign tumors (*n* = 13). LCT= Leydig cell tumor (*n* = 5, without malignancy criteria in histopathology); various benign histopathologies (*n* = 8), including status post infection (*n* = 2), normal testicular tissue (*n* = 1), status post testicular torsion (*n* = 1), simple testicular cyst (*n* = 1), epidermal cyst (*n* = 1), Sertoli cell tumor (*n* = 1), and paratesticular adenomatoid tumor (*n* = 1).

The surgical procedures performed included orchiectomy (*n* = 33; 71.7%) and partial testicular resection/testicular biopsy (*n* = 11; 23.9%). The operations were performed a median of 1 day after MRI (range 1–40 days). The distribution of histopathology can be found in Figure 4.

The median tumor size in histopathology was 2.4 cm (range 0.3–9.0) for malignant tumors and 0.8 cm (range 0.4–3.3) for benign tumors.

### 3.2. Results of MRI

Various diagnostic parameters have been employed in the assessment of tumor quality on MRI. Findings initially categorized as “questionable malignant” in the MRI assessment were reclassified as malignant, while those initially labeled as “questionable benign” were reassigned as benign.

#### 3.2.1. Tumor Signaling in Relation to Contrast Agent

The contrast agent enhancement data (type and pattern) can be found in Table 1 and Table 2.

#### 3.2.2. Diffusion Restriction

**Figure 5:**
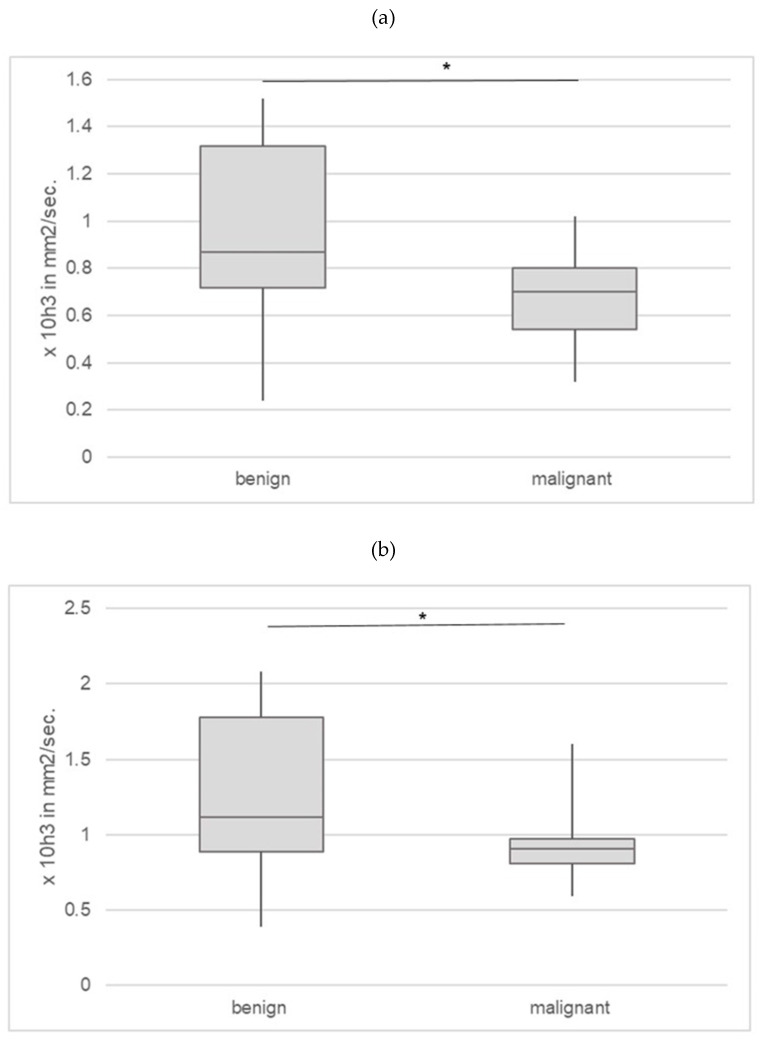
(**a**) Minimum apparent diffusion coefficient (ADC) of the lesions according to final dignity (* *p* < 0.05). (**b**) Mean apparent diffusion coefficient (ADC) of the lesions according to the final dignity (* *p* < 0.05).

The minimum ADC of the benign lesions was 0.9 (range 0.2–1.5, 50% CI 0.7–1.3), and that of the malignant lesions was 0.7 (range 0.3–1.0, 50% CI 0.5–0.8; * *p* = 0.034). The results can be found in Figure 5a.

#### 3.2.3. Morphological Characteristics of the Tumors

The correlation between the morphological characteristics of tumors on MRI and their correlation with final tumor dignity can be found in Table 2.

#### 3.2.4. Dignity Dichotomy and Number Needed to Harm (NNH)

The radiologists classified *n* = 10 (76.9%) of the benign findings as (potentially) benign and n = 3 (23.1%) as (potentially) malignant. For the malignant findings, *n* = 2 (5.7%) were assessed as (potentially) benign, while *n* = 33 (94.3%) were assessed as (potentially) malignant (*** *p* < 0.001 overall, statistical test: chi-squared test). Notably, 2/2 of the mature teratomas were incorrectly classified as benign on MRI. The number needed for harm (NNH), defined as a misdiagnosis of the MRI (false negative or false positive), correlated with the patient’s final dignity, at 9.6 (95% CI 5.3–56.4).

The sensitivity, specificity, positive predictive value (PPV), negative predictive value (NPV), efficiency, and misdiagnosis for MRI for the identification of both benign and malignant tumors based on the final histopathology were calculated for MRI. The results are provided in Table 3.

False negativity was observed in two patients, both of whom had mature teratomas. False positivity was detected in three patients—twice in LCTs and once in status post infection (Table S2).

#### 3.2.5. Correlation of Clinical Factors with Tumor Dignity

Table 4 provides a detailed comparison of clinical and diagnostic parameters for patients with benign and malignant histology. Notably, only a subset of the most important clinical parameters is presented.

In addition to the factor ‘tumor marker positive’ (*p* = 0.008), tumor size on histopathology and MRI were the only clinical factors for which benign and malignant tumors significantly differed. According to histopathology, benign tumors had a median size of 0.8 (range 0.4–3.3) cm, while malignant tumors had a median size of 2.4 (range 0.3–9.0) cm (*p* = 0.039). On MRI, benign tumors had a median size of 1.1 (range 0.5–3.7) cm, while malignant tumors had a median size of 2.0 (range 0.3–8.5) cm (*p* = 0.031).

## 4. Discussion

MRI’s role in the initial diagnosis of testicular tumors is consistently developing. Traditional US has been a well-established approach for many years; it exhibits a sensitivity ranging from 92% to 98% and a specificity ranging from 95% to 99.8%, based on the available literature [13]. While US is more cost-effective and widely available, multiparametric MRI stands out due to its ability to employ various imaging sequences. In the literature, MRI of the testis demonstrated consistently high specificity (up to 88%) and sensitivity (up to 100%) when distinguishing between benign and malignant lesions [21,25]. In our study, we demonstrated a very good sensitivity of 94.3% and a comparable specificity of 76.9%.

It has been shown that tumors of the testis exhibit distinct responses to MR contrast agents. For example, compared with seminomas, benign testicular tumors, such as Leydig cell tumors (LCTs), display higher levels of maximum relative contrast media enhancement and reach their peak enhancement more rapidly [19,26]. In a 2019 MRI study by Tsili et al. [20], four distinct patterns of enhancement were defined—type 0 (no contrast enhancement; typical for benign lesions), type I (homogeneous uptake and continuous increase in enhancement; typical for normal testicular tissue), type II (fast increase in enhancement, continued by plateauing or gradual further increase; common in benign lesions such as LCT), and type III (early, intense enhancement followed by gradual decrease; typical for malignant lesions).

In our study, a significant difference in the enhancement pattern was observed between malignant and benign tumors; the benign lesions exhibited a predominantly homogeneous uptake in 66.7% of patients and a heterogeneous uptake in only 22.2% of patients. In contrast, malignant tumors showed a predominantly heterogeneous enhancement in 62.9% of patients, and a homogeneous enhancement in only 14.3% of patients. This difference was found to be statistically significant (** *p* = 0.005), with adjusted standardized residuals for each category (>1.96 or <−1.96). Additionally, when classifying time–intensity curves according to the criteria of Tsili et al. [20,23] (as mentioned above), our study demonstrated a significant difference between benign and malignant tumors. By considering the adjusted standardized residuals, our study particularly highlighted a distinction in the Type 0 curve (no contrast enhancement); while this type of curve was observed in 25.0% of benign tumors, it was observed only in 2.9% of malignant tumors (* *p* = 0.025).

DWI with measurements of apparent diffusion coefficient (ADC) values significantly enhanced the specificity of MRI. Malignant testicular tumors seem to have significantly restricted diffusion and, thus, a reduced ADC value. In a retrospective analysis conducted by Wang et al. involving 63 testicular MR images (comprising 46 malignant and 17 benign lesions), they observed an improvement in specificity from 79.3% to 89.1% [27]. The mean or median ADC for malignant tumors typically ranges from 0.79 to 0.97, whereas for benign tumors, it falls within the range of 1.06 to 1.58 on MRI [28,29,30,31,32]. In our study, a statistically significant difference was observed between the ADC values of malignant and benign tumors. The minimum ADC value for benign lesions was 0.9, whereas for malignant lesions, it was 0.7 (each ×10^3^ mm^2^/s, *p* < 0.05). The mean ADC value for benign lesions was 1.1, and for malignant lesions, it was 0.9 (each ×10^3^ mm^2^/s, *p* < 0.05). A comparison with the data of the selected studies can be found in Table S3 [33].

Other research groups have identified additional morphological characteristics that can serve as indicators of the malignancy of testicular tumors on MRI. These characteristics include changes in T1 [25] or T2 [34] signaling compared to that in normal testicular tissue, (perfused) septa [27], hemorrhage [35], necrosis [36], calcifications [37,38], ipsilateral hydroceles [39], and regularity of tumor margins [40].

Clinical characteristics can provide hints about the malignancy of a suspected testicular tumor. Risk factors for malignant testicular tumors include age, a history of cryptorchidism or testicular cancer, testosterone deficiency, small testicular volume, and cannabis use [2,9]. However, in our study, no associations were found between any of these characteristics and tumor severity (all *p* values > 0.05). It is important to note that the subgroups with a positive history of these risk factors in our patient cohort were indeed quite small. For instance, only three patients reported cannabis consumption, seven had a history of cryptorchidism, and two had a history of testicular cancer. The only clinical parameters that demonstrated an association with the dignity of the tumor were positivity for tumor markers (** *p* < 0.01) and the size of the tumor, according to both MRI and histopathology data (* *p* < 0.05 for each parameter).

Our own research group previously presented similar results in a retrospective multi-center study of 113 MRIs of indeterminate testicular lesions. According to histopathology (*p* = 0.001), conventional US (*p* = 0.001), and MRI (*p* = 0.004), benign lesions were significantly smaller than malignant lesions. Similar findings are evident in the literature. According to Shilo et al.’s 2012 study [41], benign testicular lesions were 63.4% smaller than malignant lesions, measuring 1.5 cm and 4.1 cm, respectively. The study group set a threshold of 1.9 cm, where only 2% of findings were benign above it, compared to 38.5% below it (*p* < 0.05) [41]. The chances of larger lesions being palpable are higher, and historically, 90–95% of palpable testicular tumors have been found to be malignant [42]. Malignant testicular tumors are typically diagnosed with a median size of 3 cm (IQR 1.8–4.5 cm) [43]. In 2013, Abboudi et al. demonstrated that more than two-thirds of testicular tumors measuring less than 1 cm were benign [44].

Nonetheless, the size of a lesion does not reliably indicate its dignity, and when lesions are smaller, their classification using US alone can become more challenging. In 2018, Bieniek et al. [45] reported an incidence of 2.9% for indeterminate testicular lesions < 1 cm in a population of more than 4000 men who underwent urological consultation for fertility evaluation. Among these patients, eighteen underwent histological examination, and six were determined to have malignant tumors. However, these patients did not exhibit any significant differences in terms of size or vascularity during initial US examination compared to those of benign lesions [45].

In summary, the data from this study are in favor of MRI in the setting of testicular tumors. In the present study, we demonstrated a sensitivity of 94.3%, a specificity of 76.9%, a PPV of 91.7%, and an NPV of 89.6%. Only two patients were false negatives, and both of these patients had mature teratomas according to the final histopathology.

When the diagnosis is unclear using standard examination techniques, such as ultrasound, invasive diagnostics typically remain the only option in clinical practice, involving surgical histological sampling. In the future, if MRI becomes capable of making a safe diagnosis non-invasively, especially concerning findings that do not require surgical intervention, the invasiveness in the urological diagnostics of this condition can be significantly reduced. This not only reduces disease-related morbidity for patients, but also has the potential to lower costs; in 2014, Aberger et al. estimated the costs of an orchiectomy for ultimately benign histology, including 6–7 preparatory outpatient appointments, at Kansas Medical Center, USA to be over USD 7000 [46]. In the USA, the average cost of an MRI is around USD 1300, depending not only on the imaging modalities, but also on local circumstances [47]. 

Of course, it must also be noted that MRI availability will not be uniform for all patients worldwide, and access may vary in terms of speed. In 2008, the World Health Organization (WHO) reported that 90% of the world’s population lacked access to MRI [48]. The MRI market, however, continues to grow steadily; the global MRI market was estimated at USD 6.6 billion in 2023, with an annual growth rate of 6.5% [49].

This is a prospective observational study with inherent limitations. First, the patient cohort was relatively small and was investigated at a single academic hospital. This could influence the statistical power and the ability to detect small effects that may exist within the study population. Furthermore, the generalizability of the results to a different, broader group is certainly limited, especially those with different clinical characteristics. However, due to the low incidence of the disease in the general population, conducting prospective large-scale studies of high quality on this research question appears challenging. Nevertheless, this should help to further validate and generalize our findings. 

Moreover, all tumors that were assessed as benign upon imaging were not included in the study and were, therefore, not examined histologically. Third, ROI placement and DCE and ADC analyses may be influenced by heterogeneous tumor sizes; therefore, there may be some bias. Nevertheless, to the best of our knowledge, this is one of the largest reported prospective series of testicular tumors evaluated with DCE-MRI and DWI.

## 5. Conclusions

This represents one of the largest cohorts of a prospective, standardized MRI study described to date for the evaluation of testicular lesions, followed by histopathological examination. MRI has demonstrated excellent results in terms of sensitivity, specificity, positive predictive value (PPV), and negative predictive value (NPV) for this indication. Malignant and benign tumors can be differentiated with MRI based on the type and pattern of enhancement. Malignant tumors have significantly lower apparent diffusion coefficient (ADC) values and are significantly smaller than benign tumors. In the future, MRI could represent an important pillar in the diagnosis of testicular lesions and could enable testis-preserving strategies in a select subgroup of patients.

## Figures and Tables

**Table 1:** Properties of tumors upon contrast agent administration according to final dignity (* *p* < 0.05, ** *p* < 0.01; statistical test: Chi test). Due to the limited group size for calculating *p*-values for type and pattern of contrast agent uptake, statistical analysis could only be conducted across the entire cohort. For variables encircled in Table 2, the adjusted standardized residuals for each were >1.96 or <−1.96, indicating a high likelihood of a statistically significant difference between benign and malignant tumors.

		Benign	Malignant	*p*-Value
Type of contrast enhancement (compare [20])	*n* absence of enhancement/Type 0 curve (%)	3 (25.0)	1 (2.9)	*p* = 0.025 *
	*n* Type I curve (%)	2 (16.7)	10 (28.6)	
	*n* Type II curve (%)	2 (16.7)	17 (48.6)	
	*n* Type III curve (%)	5 (41.7)	7 (20.0)	
Pattern of contrast enhancement	*n* homogenous (%)	6 (66.7)	5 (14.3)	*p* = 0.005 **
	*n* heterogenous (%)	2 (22.2)	22 (62.9)	
	*n* rim-like (%)	1 (11.1)	8 (22.9)	

**Table 2:** Morphological characteristics of the tumors according to final dignity (* *p* < 0.05, statistical test: Mann–Whitney U-Test ^1^, Chi-test ^2^, Fisher’s exact test ^3^).

		Benign	Malignant	*p*-Value
T1 signal	*n* predominantly low (%)	2 (16.7)	3 (8.6)	0.387 ^1^
	*n* isointense (%)	10 (83.3)	30 (85.7)	
	*n* predominantly high (%)	0 (0)	2 (5.7)	
T2 signal	*n* predominantly low (%)	6 (50.0)	25 (71.4)	0.148 ^2^
	*n* isointense (%)	1 (8.3)	5 (14.3)	
	*n* predominantly high (%)	4 (33.3)	5 (14.3)	
	*n* no signal (%)	1 (8.3)	0 (0)	
Presence of enhancing intra-tumoral septa	*n* Yes (%)	2 (16.7)	17 (48.6)	*p* = 0.087 ^3^
	*n* No (%)	10 (83.3)	18 (51.4)	
Presence of areas of hemorrhage (= T1 hyperintensity)	*n* Yes (%)	0 (0)	5 (14.3)	*p* = 0.309 ^3^
	*n* No (%)	12 (100)	30 (85.7)	
Presence of areas of necrosis (= T2 hyperintensity)	*n* Yes (%)	2 (16.7)	9 (25.7)	*p* = 0.703 ^3^
	*n* No (%)	10 (83.3)	26 (74.3)	
Presence of calcification	*n* Yes (%)	1 (8.3)	4 (11.4)	*p* > 0.999 ^3^
	*n* No (%)	11 (91.7)	31 (88.6)	
Presence of ipsilateral hydrocele	*n* Yes (%)	10 (83.3)	19 (54.3)	*p* = 0.095 ^3^
	*n* No (%)	2 (16.7)	16 (45.7)	
Smooth tumor margins	*n* Yes (%)	11 (91.7)	22 (62.9)	*p* = 0.077 ^3^
	*n* No (%)	1 (8.3)	13 (37.1)	

**Table 3:** MRI’s sensitivity, specificity, PPV, and NPV for benign and malignant testicular tumor detection (CI).

Sensitivity in % (95%-CI)	94.3 (80.8–99.3)
Specificity in % (95%-CI)	76.9 (46.2–95.0)
PPV in % (95%-CI)	91.7 (77.5–98.3)
NPV in % (95%-CI)	83.3 (51.6–97.9)
Efficiency in % (95%-CI)	89.6 (77.3–96.5)
Misdiagnosis in % (95%-CI)	10.4 (3.5–22.7)

**Table 4:** Comparison of clinical and diagnostic parameters between benign and malignant histopathology (MWU = Mann–Whitney U-test; * *p* < 0.05, ** *p* < 0.01; ^1^ = Mann–Whitney U (MWU)-test, ^2^ = *t*-test, ^3^ = Fisher’s exact test).

Parameter	*p*-Value
Age in years	0.233 ^1^
Size of patient in meters	0.959 ^1^
Weight in kilograms	0.905 ^2^
BMI in kg/m^2^	0.861 ^2^
Smoking status	0.498 ^1^
Status of drug use	0.176 ^3^
St. p. cryptorchism	0.656 ^3^
St. p. testicular tumor	>0.999 ^3^
St. p. inguinal/testicular operation	0.410 ^3^
Tumor marker positive	0.008 **^3^
Presence of testosterone deficiency	>0.999 ^3^
Size of tumor in histopathology in millimeters (mm)	0.039 *^1^
Size of tumor in MRI in mm	0.031 *^1^
Size of affected testis in mm^3^ (×0.72)	0.660 ^1^

## Data Availability

The data presented in this study are available on request from the corresponding author due to ethical reasons.

## Supplementary Materials

The following supporting information can be downloaded at: https://www.mdpi.com/article/10.3390/jcm13154390/s1, Table S1: patients’ basic demographics; Table S2: Correctness of MRI according to the final histopathology. Table S3: Comparison of our study data regarding mean and minimum apparent diffusion coefficient with selected literature.

## Abbreviations

AFP	alpha-fetoprotein
BF	Blood flow
BMI	Body mass index
DWI	Diffusion-weighted imaging
EAU	European Association of Urology
FSH	Follicle-stimulation hormone
GCN(IS)	Germ cell neoplasia (in situ)
HPLAP	Human placental alkaline phosphatase
LCT	Leydig cell tumor
LDH	Lactate dehydrogenase
LH	Luteinizing hormone
MRI	Magnetic resonance imaging
NPV	Negative predictive value
ns	Not significant
NSGCT	Non-seminomatous germ cell tumor
PPV	Positive predictive value
TIN	Testicular intraepithelial neoplasia
US	Ultrasound
βhCG	Human chorionic gonadotropin
WHO	World Health Organization
