# Supplementary material for: Discriminating Malignant from Benign Testicular Masses Using Multiparametric Magnetic Resonance Imaging—A Prospective Single-Center Study
Source: J Clin Med. 2024 Jul 26;13(15):4390. doi: 10.3390/jcm13154390 (PMC11313008; doi:10.3390/jcm13154390)
Supplement: Supplementary file 1 [file jcm-13-04390-s001.zip › jcm-3097964-supplementary.pdf]

Table S1. patients' basic demographics (AFP= Alpha Fetoprotein, ßHCG= ß human chorionic gonadotropin, BMI= body mass index, CT= chemotherapy, DWI= Diffusion Weighted Imaging, FSH= Follicle-stimulating hormone, LDH= lactate dehydrogenase, LH= Luteinizing hormone, MRI= magnetic resonance imaging, TIN= Testicular intraepithelial neoplasia).

Total number of patients	*N*= 48
**Basic patient epidemiology**	
Median age in years (range)	37.5 (18-69)
n history of smoking no/yes/former (%)	15 (31.9)/4 (8.5)/ 28 (59.6)
n history of drug use no/yes Cannabis (%)	41 (93.2)/3 (6.8)
n history of testicular tumor no/yes (%)	46 (95.8)/ 2 (4.2)
n history of undescended testis no/yes (%)	41 (85.4)/7 (14.6)
n history of testicular/scrotal operations no/yes (%)	39 (81.3)/ 9 (18.8)
n presence of symptoms yes/no (%)	18 (72.0)/7 (28.0)
Type of symptoms: *n* palpable scrotal mass/ pain/infection (%)	9 (36.0)/7 (28.0)/1 (4.0)
Type of diagnosis: *n* incidental finding/self-examination/follow- up (%)	24 (50.0)/22 (45.8)/2 (4.2)
**Physical examination and ultrasound findings**	
Median BMI (range)	25.3 (20.4-43.4)
Tumor site: *n* right/left (%)	24 (51.1)/23 (48.9)
Clinical examination of the testis: *n* Tumor or swelling of the testis/normal/painful/swelling epididymis/hypotrophy testis (%)	28 (70.0)/7 (17.5)/3 (7.5)/1 2.5)/1 (2.5)
**Laboratory values preoperatively**	
Median AFP in mcg/l (range), standard value 0.5-10.0	2.7 (<1-146.0)
Median βHCG in U/l (range), standard value <1	<1 (<1-965.0)
Median HPLAP in mU/l (range), standard value < 100 mU/l	30.0 (<10.0 – 983.0)
Median LDH in U/l (range), standard value 135-225 U/l	185.0 (131.0-407.0)
n Tumor markers positive no/yes (%)	30 (65.2)/16 (34.8)
n Tumor markers positive in malignant tumors no/yes (%)	19 (54.3)/16 (45.7)
Median FSH in mu/ml (range), standard value 1.5-12.4	7.3 (<0.3-91.2)
Median LH in mU/ml (range), standard value 1.7-8.6	5.1 (<0.3-40.8)
Median testosterone in ng/ml (range), standard value 2.49-8.36	4.4 (1.2-10.2)
n presence of testosterone deficiency no/yes (%)	38 (92.7)/3 (7.3)
**Operation details**	
Type of operation: *n* orchiectomy/partial testicular resection or testicular biopsy/orchiectomy plus hernia repair (%)	33 71.7)/11 (23.9)/2 (4.4)
n fresh frozen section performed no/yes (%)	33 (68.8)/15 (31.3)
**Histopathology**	
n malignant/benign dignity (%)	(72.9)/ 13 (27.1)
Malignant: *n* seminoma/NSGCT (%)	26 (74.3)/9 (25.7)
Benign: *n* others/Leydig- cell- tumors (%)	10 (76.9)/3 (23.1)
Median tumor size in histopathology in cm (range)	1.9 (0.3-9.0)
for benign tumors	0.8 (0.4-3.3)
for malignant tumors	2.4 (0.3-9.0)
n T-status: T1/T2/T3 (%)	22 (64.7)/11 (32.4)/1 (2.9)
n Lymphovascular invasion L0/L1 (%)	29 (87.9)/4 (12.1)
n Vascular invasion V0/V1 (%)	25 (73.5)/9 (26.5)

n presence of rete testis infiltration: Yes/No (%)	15 (51.7)/14 (48.3)
n presence of ipsilateral TIN Yes/No (%)	17 (51.5)/16 (48.5)
n presence of contralateral TIN Yes/No (%)	19 (86.4)/3 (13.6)
n fresh frozen section consistent with final histopathology Yes/No (%)	14 (82.4)/3 (17.7)
n Stage of testicular cancer I/IIA/IIB/IIIA/IV (%)	30 (85.7)/1 (2.9)/2 (5.7)/1 (2.9)/ 1 (2.9)
**Data on MRI**	
Median volume of the affected testicle on MRI in ccm (range)	20.6 (6.9-215.4)
n Anatomical position of tumor on MRI: Intra-/extratesticular (%)	46 (97.9)/1 (2.1)
Median tumor size on MRI in cm (range)<br>for benign tumors<br>for malignant tumors	1.9 (0.3-8.5)<br>1.1 (0.5-3.7)<br>2.0 (0.3-8.5)
n benign/potentially benign/potentially malignant/malignant (%)	6 (12.5)/6 (12.5)/5 (10.4)/31 (64.6)ß
n Singular lesion in MRI Yes/No (%)	36 (23.4)/11 (76.6)
Following therapy	
n conservative management/platinum containing CT/radiotherapy (%)	34 (72.3)/ 10 (21.3)/ 2 (4.3)

Table S2. Correctness of MRI according to the final histopathology.

Categories according to MRI	Correct (*n*)	False (*n*)
Benign	5	1
Potentially benign	5	1
Potentially malignant	5	0
Malignant	28	3
Total	43	5

Table S3 Comparison of our study data regarding mean and minimum apparent diffusion coefficient with selected literature.

		Tsili et al. 2012 [35]	Sonmez et al. 2012 [33]	Algebally et al. 2014 [30]	Tsili et al. 2017 [32]	Deininger et al. 2024
**Included testicular lesions in n**		31	15	35	56	48
**Study design**		Retrospective	NA	Prospective	Prospective	Prospective
	**Tumor dignity**					
**Minimum ADC in *10^3 mm²/s.**	**Benign**	0.29	0.63	NA	NA	0.9
	**Malignant**	0.4	0.56	NA	NA	0.7
**Mean ADC in *10^3 mm²/s.**	**Benign**	1.56	0.93	1.58 ± 0.63	1.16 ± 0.15	1.1
	**Malignant**	0.85	0.94	0.79 ± 0.16	0.82 ± 0.31	0.9
